# Influence of Maternal Nutrition and One-Carbon Metabolites Supplementation during Early Pregnancy on Bovine Fetal Small Intestine Vascularity and Cell Proliferation

**DOI:** 10.3390/vetsci11040146

**Published:** 2024-03-23

**Authors:** Mojtaba Daneshi, Pawel P. Borowicz, Yssi L. Entzie, Jessica G. Syring, Layla E. King, Kazi Sarjana Safain, Muhammad Anas, Lawrence P. Reynolds, Alison K. Ward, Carl R. Dahlen, Matthew S. Crouse, Joel S. Caton

**Affiliations:** 1 Department of Animal Sciences, Center for Nutrition and Pregnancy, North Dakota State University, Fargo, ND 58108, USA; pawel.borowicz@ndsu.edu (P.P.B.); yssi.entzie@ndsu.edu (Y.L.E.); kazi.safain@ndsu.edu (K.S.S.); muhammad.anas.1@ndsu.edu (M.A.); larry.reynolds@ndsu.edu (L.P.R.); carl.dahlen@ndsu.edu (C.R.D.); 2 Department of Agriculture and Natural Resources, University of Minnesota Crookston, Crookston, MN 56716, USA; king0635@crk.umn.edu; 3 Department of Veterinary Biomedical Sciences, University of Saskatchewan, Saskatoon, SK S7N 5B4, Canada; alison.ward@usask.ca; 4 United States Department of Agriculture, Agriculture Research Service, U.S. Meat Animal Research Center, Clay Center, NE 68933, USA; matt.crouse@usda.gov

**Keywords:** capillary, developmental programming, fetal intestine, nutrition, one-carbon metabolism, proliferation, VEGF

## Abstract

**Simple Summary:**

Maternal nutrition during gestation can have profound effects on fetal growth and development. Perturbations to maternal nutrition during this period can predispose to adverse health and productivity outcomes in postnatal life. A biochemical pathway present in cells that can be affected by limited maternal nutrition is one-carbon metabolism. This pathway is related to epigenetics, which regulates gene expression or the turning on and off genes. The one-carbon metabolites (OCM) folate, vitamin B_12_, methionine, and choline are important compounds in this pathway. Our research aimed to evaluate the effect of maternal nutrition during early pregnancy on fetal intestinal characteristics and assess if OCM supplementation could mitigate changes due to altered maternal nutrition. We discovered that OCM supplementation in nutrition-restricted dams enhanced vascularity development signaling, decreased small intestinal weight, and optimized intestinal cell growth, potentially helping the fetus adapt to a lower nutrient supply.

**Abstract:**

To investigate the effects of nutrient restriction and one-carbon metabolite (OCM) supplementation (folate, vitamin B_12_, methionine, and choline) on fetal small intestine weight, vascularity, and cell proliferation, 29 (*n* = 7 ± 1 per treatment) crossbred Angus beef heifers (436 ± 42 kg) were estrous synchronized and conceived by artificial insemination with female sexed semen from a single sire. Then, they were allotted randomly to one of four treatments in a 2 × 2 factorial arrangement with the main factors of nutritional plane [control (CON) vs. restricted feed intake (RES)] and OCM supplementation [without OCM (−OCM) or with OCM (+OCM)]. Heifers receiving the CON level of intake were fed to target an average daily gain of 0.45 kg/day, which would allow them to reach 80% of mature BW by calving. Heifers receiving the RES level of intake were fed to lose 0.23 kg/heifer daily, which mimics observed production responses in heifers that experience a diet and environment change during early gestation. Targeted heifer gain and OCM treatments were administered from d 0 to 63 of gestation, and then all heifers were fed a common diet targeting 0.45 kg/d gain until d 161 of gestation, when heifers were slaughtered, and fetal jejunum was collected. Gain had no effect (*p* = 0.17) on the fetal small intestinal weight. However, OCM treatments (*p* = 0.02) displayed less weight compared to the −OCM groups. Capillary area density was increased in fetal jejunal villi of RES − OCM (*p* = 0.02). Vascular endothelial growth factor receptor 2 (VEGFR2) positivity ratio tended to be greater (*p* = 0.08) in villi and was less in the crypts (*p* = 0.02) of the RES + OCM group. Cell proliferation decreased (*p* = 0.02) in villi and crypts of fetal jejunal tissue from heifers fed the RES + OCM treatment compared with all groups and CON − OCM, respectively. Spatial cell density increased in RES − OCM compared with CON + OCM (*p* = 0.05). Combined, these data show OCM supplementation can increase expression of VEGFR2 in jejunal villi, which will promote maintenance of the microvascular beds, while at the same time decreasing small intestine weight and crypt cell proliferation.

## 1. Introduction

Maternal nutrition during pregnancy can profoundly affect fetal development, with lifelong effects on offspring health, productivity, and longevity later in life [1,2,3]. The early gestational period, although characterized by minimal fetal and placental growth, encompasses critical events such as fetal organogenesis and placentation [4]. Notably, even modest perturbations to maternal nutrition during this period can affect these events, potentially predisposing the offspring to adverse health and productivity outcomes later in life [1]. In fact, the nutritional plane and specific nutrient intake of the dam during pregnancy can leave lasting imprints on the fetal small intestine [5,6,7], which may persist into post-natal life [8,9]. Such changes include alterations in small intestinal length, mass [10,11], vascularity, and gene expression, which could potentially have negative effects on the offspring’s health [12,13]. The fetal intestine’s proper development is paramount, not only for nutrient absorption, digestion, and metabolism but also for immunity, production, and overall calf health and growth trajectory, and any impairment in its development may have long-term consequences for the offspring [14].

One of the core theories that underlines these effects is developmental programming, which proposes that stressors, including nutritional stress, during critical developmental windows can elicit both immediate and prolonged consequences in the offspring [15]. A principal mechanism by which maternal nutrition can affect fetal development is through epigenetic modifications [16]. Altered maternal nutrition during pregnancy has been observed to affect epigenetic mechanisms, such as DNA methylation, in the developing calf, which plays a substantial role in developmental programming [17,18]. Central to this epigenetic control is the role of one-carbon metabolites (OCM), including folate, vitamin B_12_, methionine, and choline. OCMs serve as vital epigenetic modifiers, influencing DNA methylation and thus regulating DNA and histone methyltransferases, thereby influencing the activation or suppression of gene expression [19,20,21]. Micronutrients like folate and vitamin B_12_, which are components of the one-carbon cycle, are important determinants of pregnancy outcome [22]. Nutrient restriction of heifers reduces energy and OCM substrates to the developing fetus, which may alter the fetal epigenome and lead to developmental programming events in offspring [23,24]. This implies that OCM, by influencing the activation or suppression of gene expression, can regulate the production of essential proteins through epigenetic modifications [20]. As a result, supplementing OCM during early gestation may counteract the adverse effects of dietary restriction by providing the necessary methyl donors required for epigenetic pathways and gene expression. However, while the influence of maternal nutrition on fetal intestine development has been observed, the potential mediating effects of OCM supplementation on these outcomes remain largely uncharted territory.

Therefore, the objective of this study was to determine the effects of restricted maternal nutrition during early gestation, with or without OCM supplementation, on vascularity and cell proliferation in the bovine fetal small intestine. We hypothesized that maternal nutrient restriction would reduce intestinal vascularity and proliferation, while one-carbon supplementation would at least partially ameliorate these effects. The data will provide insight into how the maternal plane of nutrition affects intestinal development in the bovine fetus and whether strategic supplementation with methyl-donor molecules may support normal gut maturation under conditions of poor maternal nutrition.

## 2. Materials and Methods

### 2.1. Animal Ethics

All procedures were approved by the North Dakota State University Animal Care and Use Committee.

### 2.2. Animals, Diet, and Treatment

Angus heifers (*n* = 72) were transported from the Central Grasslands Research Extension Center (Streeter, ND, USA) to the North Dakota State University Animal Nutrition and Physiology Center (ANPC; Fargo, ND, USA). All the heifers underwent a 7-day CO-Synch + CIDR estrus synchronization protocol [25] and were artificially inseminated 18 to 22 h after detected estrus. Since fetal sex and maternal nutrition significantly affect the development of bovine fetuses during gestation [26,27], female-sexed semen from a single sire (Connealy Maternal Made [ST Genetics, Navasota, TX, USA]) was used. Out of these, 32 heifers (8 per group, average initial body weight (BW) = 436 ± 42 kg) became pregnant.

At breeding, the heifers were divided into four nutritional treatments in a 2 × 2 factorial arrangement with the main factors of the nutritional plane (control vs. restricted feed intake) and OCM supplementation (without OCM; −OCM; or with OCM; +OCM). Each heifer was fed daily using an electronic head gate system at the facility (provided by American Calan; Northwood, NH, USA).

Heifers receiving the control (CON) level of intake were fed 100% of the NASEM [28] requirements to target an average daily gain (ADG) of 0.45 kg/day (actual: 0.60 kg/d ADG), which would allow them to reach 80% of mature BW by calving. Heifers receiving the restricted (RES) level of intake were fed to lose 0.23 kg/heifer daily (actual: −0.23 kg/d ADG), which mimics observed production responses in heifers that experience a diet and environment change during early gestation [28]. Diets were a total mixed ration of corn silage, alfalfa hay, corn grain, alfalfa/grass hay, and vitamin/mineral premix (Trouw dairy VTM w/Optimins, Trouw Nutrition USA, Highland, IL, USA) and were fed to reach the targeted gains described above. Heifers were weighed, and diet adjustments were made weekly to reach the targeted rates of gain.

The OCM-supplemented heifers (+OCM) received 7.4 g/d of rumen-protected methionine (Smartamine, Adisseo, Beijing, China) and 44.4 g/d of rumen-protected choline (ReaShure, Balchem Inc., New Hampton, NY, USA) in a corn carrier, with the dosages used in previously published research [29,30]. Additionally, these heifers received weekly intramuscular injections of 20 mg of vitamin B_12_ (MWI Animal Health, Boise, ID, USA) plus 320 mg of folic acid (Spectrum Chemical Mfg. Corp., New Brunswick, NJ, USA), as described in earlier studies [29,31]. The heifers that were not supplemented with OCM (−OCM) received the corn carrier in their feed daily, along with weekly intramuscular saline injections). Treatments were provided until day 63 of gestation, after which all heifers were transitioned to the CON − OCM treatment for a target rate of gain of 0.45 kg/d for the remainder of the study. Pregnancy was verified on day 35 of gestation through transrectal ultrasonography by observing the fetal heartbeat. Ultrasound evaluations were subsequently performed on day 63 to determine the sex of the fetuses [32], and only female fetuses were included in the later stages of the project.

### 2.3. Sample Collection and Preparation

On day 161 of gestation, heifers were slaughtered. Samples were collected from the fetal jejunum and preserved in 4% neutral buffered formalin (NBF). The sampling location was determined by following the third branch of the mesenteric vein caudal to the portal vein, then tracking up the venous arcade to the point of insertion into the jejunal tissue, and then removing a jejunal sample caudal to this location [5]. Tissues were fixed for 24 h in NBF and then placed in 70% ethanol. For histopathological examination, the tissues were dissected as cross sections of 2–3 mm thickness, dehydrated in ascending grades of alcohol, cleared in xylene, infiltrated with paraffin wax, and finally embedded using Tissue-Tek TEC (Sakura^©^).

### 2.4. Immunohistochemistry and Immunofluorescence

Tissues were sectioned to a thickness of 5 μm for immunostaining. Each slide was deparaffinized in xylene and rehydrated through a graded alcohol series. For antigen retrieval, sections were heated in 0.01 M sodium citrate buffer (pH 6) using a pressure cooker for 20 min, then cooled to room temperature for 10 min and washed with TBST for 3 min. Subsequently, to block nonspecific antibody binding, the sections were incubated with 5% normal goat serum for 1 h at room temperature (approximately 23 °C). The tissue sections were then incubated with primary antibodies, including anti-cluster of differentiation (CD31) (1:50 ab28364, Abcam, Boston, MA, USA ) and anti-CD34 (1:500, ab81829, Abcam) to identify vascular endothelial cells, anti-Ki-67 (1:500, ab15580, Abcam) to identify proliferating cells, and vascular endothelial growth factor receptor 2 (VEGFR2) (1:250, ab2349, Abcam) to identify VEGF-responsive cells for 1 h at room temperature in a humidified chamber, except for VEGFR2, which was incubated overnight at 4 °C. Following primary antibody incubation, sections were washed with TBST for 3 min. The sections were then incubated with an Alexa Fluor™ 633 goat anti-rabbit secondary antibody for 1 h at room temperature. Finally, nuclei were counterstained with 4′,6-diamidino-2-phenylindole (DAPI 1%, BIOTIUM, Fremont, CA, USA) for 5 min.

### 2.5. Image Processing

Images were captured from slides using two fluorescence microscopes: the Zeiss LSM700 laser scanning confocal microscope (Zeiss, Oberkochen, Germany) with a 20× 0.8 NA objective for CD31 and CD34, and a 10× 0.8 NA objective for Ki-67; and the Leica Mica (Leica, Germany) in thunder mode with a PL APO 10× 0.75 NA objective for VEGFR2. The acquired images were then subjected to detailed analysis using Image Pro-Premier software v. 9.1 (Media Cybernetics, Rockville, MD, USA).

**Figure 1:**
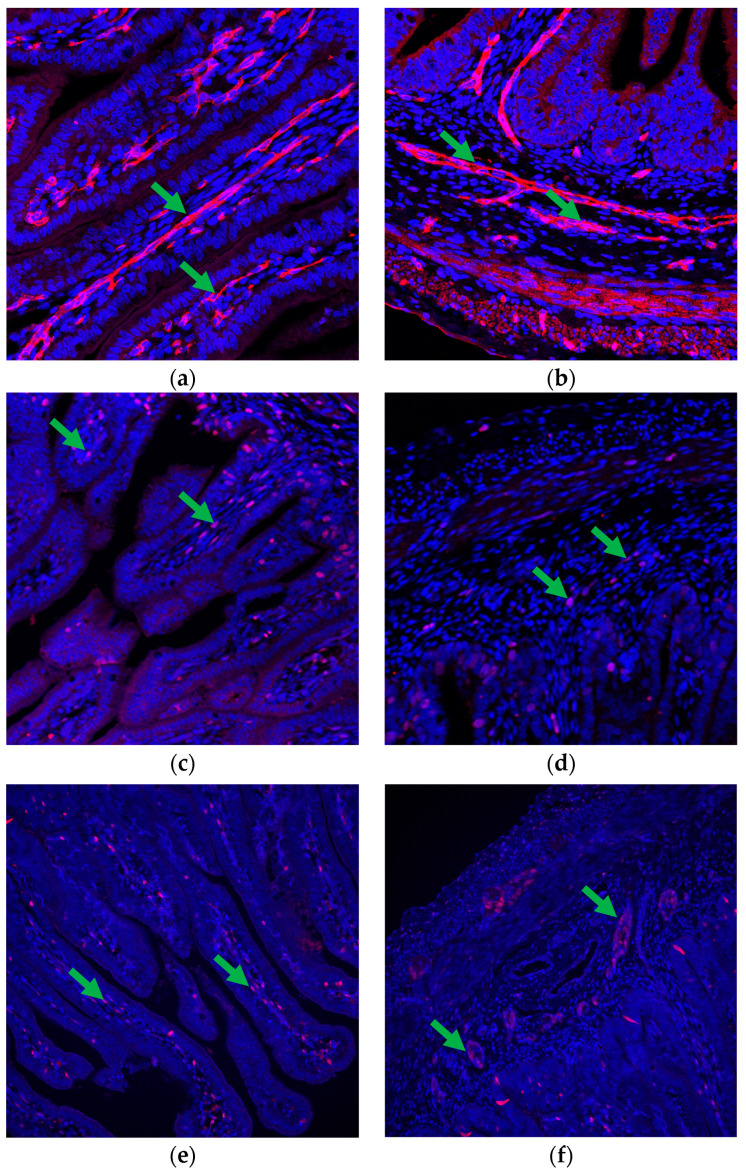
Vascularity and cell proliferation in the fetal small intestine. (**a**,**b**) Immunofluorescence staining highlights the presence of positive CD31 + CD34 cells (indicated by green arrows) in both the (**a**) villi and (**b**) crypts, demonstrating the presence of capillaries within these structures and indicating vascular development. (**c**,**d**) Immunofluorescence staining using anti-Ki-67 antibodies reveals positive Ki-67 cells (marked with green arrows) in both the (**c**) villi and (**d**) crypts, indicating active cell proliferation in these regions. (**e**,**f**) Immunofluorescence staining with anti-VEGFR2 antibodies highlights the presence of positive VEGFR2 cells (indicated by green arrows) in both (**e**) the villi and (**f**) crypts, suggesting responsiveness to VEGF signaling and potential involvement in vascular development and maintenance. CD31 + CD34 images are at 200× magnification, whereas Ki-67 and VEGFR2 images are at 100× magnification.

Specific quantitative analyses [9,11,13] were conducted on the immunofluorescence images obtained from the small intestine slides. These images, stained with targeted antibodies, were analyzed to calculate various factors indicative of intestinal vascularity, cell proliferation, and protein expression. Capillary area density (CAD) (Figure 1a,b) was determined by dividing the selected area of CD31 plus CD34 staining by the total visible tissue area and multiplying the result by 100. Capillary number density (CND) was calculated by dividing the number of selected vascular (CD31- plus CD34-positive) elements by the total tissue area and multiplying the result by 10,000. The positivity ratio of Ki-67 (KPR) (Figure 1c,d) was determined by dividing the total tissue area of positive Ki-67 cells by the total cells and multiplying the result by 100. Spatial cell density (SCD) was calculated by dividing the total cells by the total tissue area and multiplying the result by 10,000. The VEGFR2 positivity ratio (VPR) (Figure 1e,f) was determined by dividing the area of positive VEGFR2 cells by the area of total cells and multiplying the result by 100. The spatial VEGFR2 positivity rate (SVPR) was determined by dividing the area of positive VEGFR2 cells by the total tissue area and multiplying the result by 10,000.

### 2.6. Statistical Analysis

The data were subjected to statistical analysis using PROC MIXED of SAS v.9.4 software (SAS Institute Inc., Cary, NC, USA). All variables were initially assessed for normality of distribution using PROC UNIVARIATE, followed by the qqplot statement. If the normality assumption was not met, the data were log-transformed, and the test for normality was repeated using the same procedure. The fixed effects in the model included the nutritional plane, OCM treatment, and their interaction, with each individual heifer serving as the experimental unit. If no significant interactions were observed, the main effects of the maternal nutritional plane and OCM treatment were reported. Data are presented as the least squares mean and standard error of the mean (SEM), and any transformed data were reverted to their original values after analysis. The largest SEM is reported. Pearson correlation coefficients between variables were also examined using the PROC CORR procedure. All plots were created using ggplot2 v.3.4.1 in R Studio v.4.2.2. Statistical significance was declared at a *p*-value ≤ 0.05, and tendencies were reported for 0.05 < *p* ≤ 0.10.

## 3. Results

One fetus in each of the CON + OCM, RES − OCM, and RES + OCM groups was male, and was subsequently excluded from the study.

### 3.1. Small Intestine Phenotype

In evaluating the effects of maternal nutrition plane (CON vs. RES) and one-carbon metabolite supplementation (−OCM vs. +OCM) on the physical development of the bovine fetal small intestine, the study measured the small intestine weight (SIW) and its ratio to the body weight across the four treatment groups (Table 1). No interaction between maternal intake and OCM supplementation (*p* = 0.47) was observed in the SIW. However, the +OCM groups (64.08 ± 2 g) displayed a lower (*p* = 0.02) SIW compared to the −OCM groups (71.08 ± 2.06 g). Furthermore, there was a tendency (*p* = 0.08) for both maternal intake and OCM to influence the ratio of SIW to body weight. The RES groups exhibited a lower ratio (1.68 ± 0.05 g/kg) compared to the CON groups (1.83 ± 0.05 g/kg), and similarly, the +OCM groups showed a lower ratio (1.68 ± 0.05 g/kg) compared to the −OCM groups (1.83 ± 0.05 g/kg).

### 3.2. Capillary Development of the Fetal Small Intestine

**Figure 2:**
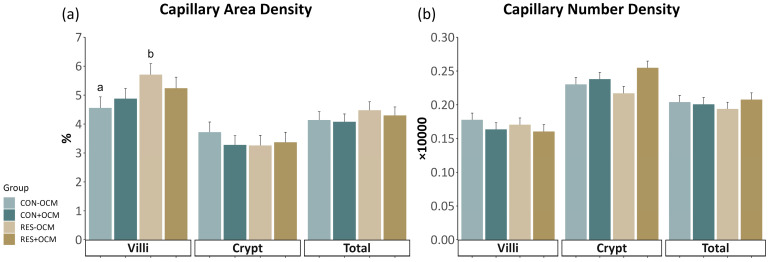
Capillary development in the fetal small intestine: (**a**) capillary area density (CAD) and (**b**) capillary number density (CND) across villi, crypts, and total intestinal area. Different lowercase letters indicate *p* ≤ 0.05.

The effect of maternal nutrition plane (CON vs. RES) and one-carbon metabolite supplementation (−OCM vs. +OCM) during early gestation on the capillary development of the fetal intestine was assessed through measurements of capillary area density (CAD) and capillary number density (CND) in the villi, crypts, and total intestine on gestational day 161 (Figure 2; Supplementary Table S1). In the villi, no interaction between maternal intake and OCM supplementation (*p* = 0.29) was observed for CAD. However, there was a significant effect of maternal intake on CAD (*p* = 0.05), with CAD being increased by 16% in the RES groups (5.47 ± 0.27%) compared to the CON groups (4.72 ± 0.26%). The CAD and CND values across the crypts (*p* = 0.42, *p* = 0.33, respectively) and the total intestine (*p* = 0.12, *p* = 0.4, respectively) did not show significant differences among the groups.

### 3.3. VEGFR2 Expression in the Fetal Small Intestine

**Figure 3:**
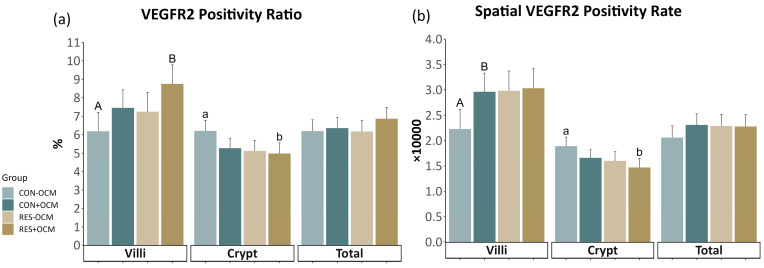
VEGFR2 expression in the fetal small intestine: (**a**) VEGFR2 positivity ratio (VPR) and (**b**) spatial VEGFR2 positivity rate (SVPR) across villi, crypts, and total intestinal area. Lowercase letters indicate significant differences (*p* ≤ 0.05), and uppercase letters denote trends (0.05 < *p* ≤ 0.10).

Vascular endothelial growth factor receptor 2 (VEGFR2) expression was evaluated using the VEGFR2 positivity ratio (VPR) and spatial VEGFR2 positivity rate (SVPR), as shown in Figure 3 (Figure 3; Supplementary Table S2). No interactions between maternal intake and OCM supplementation or main effects were observed in any of the evaluated areas. However, in the villi, a tendency was noted (*p* = 0.08) for a greater VPR in the +OCM groups (8.1 ± 0.71 × 10,000) compared to the −OCM groups (6.72 ± 0.73 × 10,000).

A significant difference (*p* = 0.05) was observed in the crypt VPR due to maternal intake, with the RES groups exhibiting lower values (5.05 ± 0.41 × 10,000) compared to the CON groups (5.74 ± 0.4 × 10,000). Additionally, a significant difference (*p* = 0.02) was found in the crypt SVPR due to maternal intake, with the RES groups showing lower values (1.53 ± 0.12 × 10,000) compared to the CON groups (1.77 ± 0.12 × 10,000). No significant differences in VPR (*p* = 0.4) and SVPR (*p* = 0.35) were observed in the total area across any of the treatments.

### 3.4. Cell Proliferation of the Fetal Small Intestine

**Figure 4:**
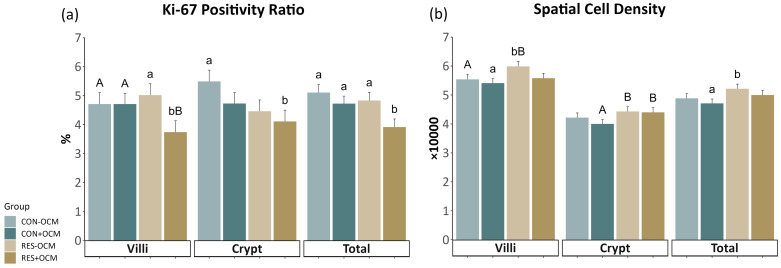
Proliferation of the fetal small intestine: (**a**) Ki-67 positivity ratio (KPR) and (**b**) spatial cell density (SCD) across villi, crypts, and total intestinal area. Lowercase letters indicate significant differences (*p* ≤ 0.05), and uppercase letters denote trends (0.05 < *p* ≤ 0.10).

Cell proliferation in the fetal intestine was assessed by measuring the Ki-67 positivity ratio (KPR) and spatial cell density (SCD) (Figure 4; Supplementary Table S3). In the villi, there was a tendency for an interaction (*p* = 0.09) on KPR, where the RES + OCM group had significantly lower values compared to the RES − OCM group (*p* = 0.02) and tended to have lower values than both CON − OCM (*p* = 0.08) and CON + OCM (*p* = 0.07) groups. Additionally, a tendency (*p* = 0.09) was observed for OCM on KPR in the villi, with the +OCM groups (4.21 ± 0.27%) showing lower values than the −OCM groups (4.87 ± 0.28%). For SCD in the villi, a tendency (*p* = 0.08) was noted for the effect of nutritional intake, with the RES groups (5.79 ± 0.12 × 10,000) having greater values than the CON groups (5.48 ± 0.12 × 10,000).

In the crypts, both maternal intake (*p* = 0.06) and OCM supplementation (*p* = 0.1) tended to affect KPR. Here, the RES groups (4.37 ± 0.28%) had lower values than the CON groups (5.11 ± 0.27%), and the groups receiving OCM (4.42 ± 0.27%) had lower values than those not receiving OCM (5.06 ± 0.28%). Similarly, maternal intake showed a tendency (*p* = 0.06) to affect SCD in the crypts, with the RES groups exhibiting higher values (4.42 ± 0.11 × 10,000) than the CON groups (4.11 ± 0.11 × 10,000).

No significant interaction between maternal intake and OCM supplementation was observed in the total intestinal area. However, both maternal intake (*p* = 0.05) and OCM supplementation (*p* = 0.01) had separate effects on KPR. The RES groups exhibited lower values (4.37 ± 0.19%) compared to the CON groups (4.91 ± 0.19%), and the +OCM groups (4.32 ± 0.19%) had lower values than the −OCM groups (4.97 ± 0.19%). Similarly, maternal intake (*p* = 0.05) had an effect on SCD in the total area, where the RES groups (5.1 ± 0.11 × 10,000) showed higher values compared to the CON groups (4.79 ± 0.11 × 10,000).

### 3.5. Correlations between Variables

The Pearson correlation coefficients were calculated among various variables (VPR, SVPR, KPR, SCD, CAD, CND, and SIW) in the total area for each treatment group (Supplementary Table S4). In the CON − OCM group, a positive correlation was observed between VPR and CAD (*p* = 0.04), and a positive correlation was noted between SVPR and CAD (*p* ≤ 0.01). The CON + OCM group exhibited a range of correlations. The variable VPR showed a positive correlation with SCD (*p* = 0.04) and a positive correlation with CAD (*p* ≤ 0.01), while also presenting a positive trend with SIW (*p* = 0.06). SVPR displayed positive correlations with both SCD (*p* ≤ 0.01) and CAD (*p* ≤ 0.01) and a negative correlation with CND (*p* ≤ 0.01). Additionally, SCD correlated positively with CAD (*p* ≤ 0.01) and negatively with CND (*p* ≤ 0.01), and CAD showed a negative correlation with SIW (*p* = 0.05). In the RES − OCM group, KPR correlated positively with both CAD (*p* ≤ 0.01) and SIW (*p* ≤ 0.01) and with CND (*p* = 0.05). Also, SCD showed a positive correlation with CAD (*p* ≤ 0.01) and a negative correlation with CND (*p* = 0.04). Finally, the RES + OCM group demonstrated a positive correlation between VPR and SCD (*p* ≤ 0.01), and a positive correlation with CAD (*p* = 0.04). The SVPR correlated positively with SCD (*p* ≤ 0.01), with CAD (*p* ≤ 0.01), and showed a negative trend with CND (*p* = 0.06). Additionally, KPR showed a positive correlation with SIW (*p* ≤ 0.01), and SCD exhibited positive correlations with CAD (*p* = 0.02) and negative correlations with CND (*p* ≤ 0.01).

## 4. Discussion

The present study aimed to elucidate the impacts of restricted maternal nutrition during early gestation, with or without OCM supplementation, on phenotype, vascularity, and cell proliferation in the bovine fetal small intestine. We hypothesized that nutrient restriction would reduce intestinal development, while OCM supplementation would ameliorate some of these effects. In alignment with our hypothesis, maternal nutrient restriction during the first two months of gestation did appear to influence fetal intestinal morphology later in pregnancy.

### 4.1. Fetal Small Intestine Weight

The SIW showed no notable differences between the CON and RES groups, suggesting that nutrient restriction during early gestation did not impact the SIW. This observation aligns with our previous studies, which demonstrated that maternal nutrient restriction during early to mid-gestation did not affect SIW or its body weight ratio in bovine fetuses [5,7,26], calves [8], ovine fetuses [33], and offspring in sheep [9,13]. These findings are also consistent with Duarte et al. (2013), who investigated the effect of maternal nutrition on gastrointestinal tract development in feed-restricted and ad libitum-fed Nellore cows, finding no significant differences in the mass of the fetal small intestine at various stages of gestation [34]. Recent work by Zhang et al. (2021) further supports our findings, concluding that low levels of energy intake throughout gestation in cattle do not impact fetal SIW [35].

It has been demonstrated that the intestinal development of bovine fetuses during gestation is affected by fetal sex, where female fetuses born to dams under restricted nutrition during pregnancy had greater small intestine mass and small intestine mass in proportion to body mass compared to male fetuses [26]. The mechanisms behind these observations remain unclear, but we hypothesize that female fetuses may be less responsive to maternal nutritional restriction due to evolutionary adaptations, as it has previously been hypothesized that parents in certain conditions might preferentially invest in offspring of one sex over the other based on which sex has a higher potential reproductive benefit under those conditions [36]. It has also been suggested that enhanced female intestinal development may be correlated with milk production later in life [26]. This hypothesis warrants further investigation to understand the evolutionary and physiological nuances of fetal development in livestock.

Contrary to our hypothesis, however, we observed a significant effect of maternal OCM supplementation during gestation on lowering fetal small intestine weight in both restricted and control heifers. Our laboratory has previously illustrated that OCM supplementation can increase bovine embryonic cell growth and proliferation in vitro due to greater mitochondrial efficiency [37]. In the context of our findings, if the improved efficiency observed in vitro was also present in the cells of the developing fetuses, this improved efficiency could lead to a more optimized growth trajectory, potentially resulting in a reduction in SIW as part of a broader adaptation to the altered metabolic environment induced by OCM supplementation. This adaptation might allow the animal to survive on a lower nutrient supply [11]. Additionally, the interplay between OCM and energy metabolism is likely a significant factor. The effects of OCM supplementation seem to depend on energy availability, with significant differences observed between low and high energy conditions in cell culture studies [37,38]. This suggests that the energy context provided by maternal nutrition might influence the effects of OCM supplementation on fetal development, including SIW, although our phenotype results did not show an interaction between maternal intake and OCM supplementation. Further evaluation of tissues and molecular-level responses is needed to determine if OCM supplementation is sensitive to the energy availability to the dam and how this affects the developmental programming of SIW. Moreover, the sex-specific responses to maternal nutrition suggest that the effects of OCM supplementation on fetal development might differ between male and female offspring, reflecting variations in how male and female fetuses respond to maternal nutrition, energy supply, and epigenetic modifications [21]. Given the critical role of OCM in various metabolic pathways and their involvement in epigenetic pathways [19,20], it is plausible that OCM supplementation during early gestation could have lasting effects on the developmental programming of SIW, though the underlying mechanisms remain to be elucidated.

### 4.2. Vascular Development in the Fetal Intestine

Regarding vascularity, our study reveals that early gestation maternal nutrition significantly influences capillary development, as evidenced by the increased CAD in the villi of fetuses subjected to maternal nutrient restriction later in pregnancy. This finding aligns with Meyer et al.’s research [5], which showed enhanced total vascularity in the bovine fetal small intestine due to restricted maternal nutrition. However, studies conducted in the ovine model illustrated that restricted maternal nutrition resulted in reduced vascularity in the small intestine of fetuses [11,12] and offspring [13]. The increased vascularity noted in our study likely reflects a compensatory and adaptive mechanism, potentially enhancing absorption and maintaining oxygen and nutrient supply to the developing intestine.

Our findings also indicate that the maternal nutrition plane does not significantly affect the expression of VEGFR2. This observation is consistent with our previous studies, which demonstrated no notable effect of maternal nutrient restriction on the jejunal expression of VEGF and its receptors [8,13]. It is widely recognized that sub-optimal micronutrient levels, such as folate and vitamin B_12_, involved in the one-carbon cycle, can lead to increased homocysteine levels and oxidative stress [22]. Our lab previously reported greater serum homocysteine concentrations in nutrient-restricted pregnant heifers [23] and a negative correlation between maternal serum homocysteine and fetal amniotic fluid folate [39], suggesting disrupted one-carbon metabolism caused by restricted maternal nutrition. Considering that homocysteine reduces expression of VEGF and its receptors [40,41,42], we hypothesize that VEGF and its receptors might not significantly contribute to the observed increase in CAD in fetuses born to restricted dams. Vascularization is a complex process regulated by multiple growth factors [43], and while VEGF is a crucial angiogenic factor, other mechanisms may be involved in the heightened vascularization in response to maternal nutritional restriction during pregnancy.

Interestingly, the trends in VEGFR2 expression, specifically the increased VPR in the villi of the +OCM group, suggest that OCM plays a significant role in modulating angiogenesis in a region-specific manner. The pronounced response of VEGFR2 to OCM in the villi, a region crucial for nutrient absorption, may highlight the targeted impact of OCM on areas critical for nutrient uptake. Furthermore, the OCM groups exhibited negative correlations between angiogenesis indexes (VPR and SVPR) and CND, yet positive relationships for the same factors with CAD (Supplementary Table S4). The observed trends imply that capillaries are responding more robustly to preserved angiogenesis factors in the OCM groups. This response is potentially manifested in the capillary structure, as reflected by the decrease in CND and the increase in CAD. This suggests that VEGFR2 expression may be a critical mediator in the development of capillaries in response to modifications in the maternal diet, specifically with OCM supplementation. This suggestion is in line with the discussion above on the altered one-carbon cycle and its effects on oxidative stress and angiogenesis [22]. Recent findings that OCM has antioxidant functions in the small intestine of cattle [44] support the hypothesis that OCM supplementation could potentially lead to increased VEGF and, consequently, vascularity indexes. While the direct effects of and upregulation of VEGFR2 were not immediately apparent on CAD and CND at the gestational stage examined (day 161), these changes might become more pronounced as pregnancy advances and fetal development continues. Previous studies have shown that the day of gestation has a significant interaction with maternal nutrient restriction on fetal intestinal vascularity, with bovine fetal jejunal CAD being greater at day 245 than day 125 [5]. Additionally, rapid growth and maturation of the fetal small intestine occur in mid- to late gestation, immediately before birth [14], notably influencing the expression of VEGF [45].

### 4.3. Cell Proliferation in the Fetal Intestine

In contrast to previous studies where jejunal cell proliferation was found to be reduced in lambs born to nutrient-restricted dams [9,46] or increased in the case of bovine fetuses [5], our observations indicate that the maternal nutritional plane had no observed effect on KPR, which serves as a cell proliferation index. These findings are consistent with previous research [8]. However, fetuses born to dams under restricted nutrition conditions exhibited greater SCD compared to their counterparts. Our laboratory has previously demonstrated that fetal jejunal protein: DNA ratios were lower in fetuses from nutrient-restricted ewes compared to those from control ewes, indicating smaller cell size in the fetal jejunum of restricted ewes [11]. This suggests that, in response to restricted nutrition, the gut may employ a compensatory mechanism by increasing the number of cells, which leads to an increase in the surface area-to-mass ratio, thereby enhancing nutrient absorption.

The reduction in KPR observed in the +OCM groups relative to the −OCM groups suggests a modulating effect of OCM on cell turnover. While decreased KPR in the control group might indicate impaired cell proliferation, the decline in the RES groups could alternatively reflect enhanced efficiency, with fewer cell replications or apoptotic events when methyl donors are available to facilitate proper development. This interpretation is supported by positive correlations between KPR and SIW observed only in the RES + OCM group, suggesting that methyl donors optimize growth trajectories to match available resources. A study conducted by Seyyedin and Nazem in 2017 reported that the depth of crypts in the duodenum and jejunum significantly decreased in rats receiving supplemental methionine [47]. Another study showed that feeding methionine-supplemented diets to mice resulted in a reduction in crypt depth and a decrease in the total enterocyte mass in the proximal jejunum [48]. Additionally, rat pups from dams subjected to a methyl-deficient diet (lacking vitamin B_12_, folate, and choline) exhibited increased crypt apoptosis and a loss of enterocyte differentiation in the villus [49]. Therefore, rather than disrupting signals, OCM supplementation may enable efficient tissue maturation focused on optimizing metabolic efficiency rather than on maximizing growth alone. This could represent an adaptive response that spares limited nutrients for critical developmental tasks, resulting in a slower turnover rate of the intestinal epithelium, lower maintenance requirements, and potentially greater growth efficiency in the animal [50]. The observed patterns in fetuses born to nutrient-restricted dams imply the lasting effects of poor maternal nutrition on tissue structure and function. This finding aligns with the broader concept of developmental programming, where early nutritional experiences can shape organ development and function. However, through the regulation of cell turnover and apoptosis, OCM may mitigate these effects. Given the vital epigenetic roles of methyl donors, optimized proliferation levels with OCM supplementation suggest improved energetic efficiency and controlled tissue growth within the constraints of available resources [21].

## 5. Conclusions

In conclusion, our study reveals that OCM supplementation significantly modulates vascular development in the fetal small intestines of bovine fetuses under maternal nutrient restriction, particularly affecting villi in a region-specific manner. Interestingly, our findings suggest that VEGF and its receptors may not be major contributors to the increase in vascularity in these fetuses born to dams under restricted nutrition. Additionally, OCM appears to play a crucial role in optimizing intestinal health by reducing small intestine weight and modulating cell turnover, thus enhancing the efficiency of intestinal growth. The study indicates the importance of further investigation into the progressive effects of OCM on fetal vascularity and cell proliferation during pregnancy. Additionally, more research is necessary to understand the long-term impact of OCM supplementation on offspring health.

## Figures and Tables

**Table 1:** The effect of maternal nutritional levels and supplementation with one-carbon metabolites ^1^ from day 0 to 63 of gestation on the phenotype of the fetal small intestine at 161 days of age.

	Treatments ^2^					*p*-Value ^3^		
Traits	CON − OCM	CON + OCM	RES − OCM	RES + OCM	SEM ^4^	MFI	OCM	MFI × OCM
SIW (g) ^5^	74.14	65.0	68.01	63.11	2.29	0.17	0.02	0.47
Relative SIW (g/kg of body weight)	1.89	1.77	1.77	1.59	0.08	0.08	0.08	0.75

The data are presented as the least squares mean and the standard error of the mean. ^1^ Methionine, choline, folate, and vitamin B_12_. ^2^ CON − OCM = control (0.45 kg/d) without one-carbon metabolite supplementation; CON + OCM = Control (0.45 kg/d) with one-carbon metabolite supplementation; RES − OCM = restricted (−0.23 kg/d) without supplementation; RES + OCM = restricted (−0.23 kg/d) with supplementation. ^3^ MFI = main effect of feed intake levels; OCM = main effect of one-carbon metabolite supplementation; MFI × OCM = main effect of feed intake levels interaction with one-carbon metabolite supplementation. ^4^ SEM = standard error of the mean. ^5^ Small intestine weight.

## Data Availability

The data presented in this study are available on request from the corresponding author.

## Supplementary Materials

The following supporting information can be downloaded at: https://www.mdpi.com/article/10.3390/vetsci11040146/s1, Table S1: The effect of maternal nutritional levels and supplementation with one-carbon metabolites from day 0 to 63 of gestation on the capillaries of the fetal small intestine at 161 days of age.; Table S2: The effect of maternal nutritional levels and supplementation with one-carbon metabolites from day 0 to 63 of gestation on the VEGFR2 expression of the fetal small intestine at 161 days of age.; Table S3: The effect of maternal nutritional levels and supplementation with one-carbon metabolites from day 0 to 63 of gestation on the proliferation of the fetal small intestine at 161 days of age.; Table S4: Pearson correlation coefficients for relationships between measured factors in fetal small intestine at 161 days of age.
