# Supplementary material for: Influence of Maternal Nutrition and One-Carbon Metabolites Supplementation during Early Pregnancy on Bovine Fetal Small Intestine Vascularity and Cell Proliferation
Source: Vet Sci. 2024 Mar 23;11(4):146. doi: 10.3390/vetsci11040146 (PMC11054626; doi:10.3390/vetsci11040146)
Supplement: Supplementary file 1 [file vetsci-11-00146-s001.zip › vetsci-2904127-supplementary.pdf]

Table S1. The effect of maternal nutritional levels and supplementation with one-carbon metabolites[1] from day 0 to 63 of gestation on the capillaries of the fetal small intestine at 161 days of age.

		**Treatments[2]**					***p*-value[3]**		
The evaluated area		CON-OCM	CON+OCM	RES-OCM	RES+OCM	SEM[4]	MFI	OCM	MFI×OCM
Villi	CAD[5]%	4.56	4.88	5.71	5.24	0.38	0.05	0.84	0.29
	CND[6]	0.18	0.16	0.17	0.16	0.01	0.58	0.19	0.82
Crypt	CAD%	3.72	3.28	3.26	3.37	0.35	0.59	0.62	0.42
	CND	0.23	0.23	0.21	0.25	0.01	0.90	0.12	0.31
Total	CAD%	4.14	4.08	4.48	0.29	0.33	0.67	0.83	0.33
	CND	0.20	0.20	0.19	0.01	0.87	0.60	0.40	0.87

The data are presented as the least square mean and standard error of the mean. [1]Methionine, choline, folate, vitamin B_{12}. [2]CON-OCM = Control (0.45 kg/d) without one-carbon metabolite supplementation; CON+OCM = Control (0.45 kg/d) with one-carbon metabolite supplementation; RES-OCM = Restricted (-0.23 kg/d) without supplementation; RES+OCM = Restricted (-0.23 kg/d) with supplementation. [3]MFI = Main effect of feed intake levels; OCM = Main effect of one-carbon metabolite supplementation; Gain×SUP = Main effect of feed intake levels interaction with one-carbon metabolite supplementation. [4]SEM = Standard error of the mean. [5]Capillary area density.[6]Capillary number density×10000.

Table S2. The effect of maternal nutritional levels and supplementation with one-carbon metabolites[1] from day 0 to 63 of gestation on the VEGFR2 expression of the fetal small intestine at 161 days of age.

		**Treatments[2]**					***p*-value[3]**		
The evaluated area		CON-OCM	CON+OCM	RES-OCM	RES+OCM	SEM[4]	MFI	OCM	MFI×OCM
Villi	VPR[5]%	6.19	7.46	7.25	8.75	1.03	0.42	0.08	0.82
	SVPR[6]	2.23	2.96	2.98	3.08	0.39	0.39	0.16	0.32
Crypt	VPR %	6.21	5.27	5.12	4.98	0.58	0.05	0.16	0.89
	SVPR	1.89	1.66	1.6	1.47	0.18	0.02	0.24	0.87
Total	VPR %	6.2	6.36	6.18	6.87	0.61	0.4	0.98	0.95
	SVPR	2.06	2.31	2.29	2.28	0.23	0.35	0.91	0.46

The data are presented as the least square mean and standard error of the mean. [1]Methionine, choline, folate, vitamin B_{12}. [2]CON-OCM = Control (0.45kg/d) without one-carbon metabolite supplementation; CON+OCM = Control (0.45 kg/d) with one-carbon metabolite supplementation; RES-OCM = Restricted (-0.23 kg/d) without supplementation; RES+OCM = Restricted (-0.23 kg/d) with supplementation. [3]MFI = Main effect of feed intake levels; OCM = Main effect of one-carbon metabolite supplementation; Gain×SUP = Main effect of feed intake levels interaction with one-carbon metabolite supplementation. [4]SEM = Standard error of the mean. [5]VEGFR2 positivity ratio.[6]Spatial VEGFR2 positivity rate ×10000.

Table S3. The effect of maternal nutritional levels and supplementation with one-carbon metabolites[1] from day 0 to 63 of gestation on the proliferation of the fetal small intestine at 161 days of age.

The evaluated area		Treatments[2]				SEM[4]	*p*-value[3]		
		CON-OCM	CON+OCM	RES-OCM	RES+OCM		MFI	OCM	MFI×OCM
Villi	KPR[5]%	4.71[A]	4.71[A]	5.02[a]	3.74[bB]	0.39	0.38	0.09	0.09
	SCD[6]	5.54	5.41	5.99	5.58	0.17	0.08	0.12	0.44
Crypt	KPR %	5.49	4.73	4.46	4.11	0.39	0.06	0.1	0.76
	SCD	4.22	4.00	4.43	4.4	0.16	0.06	0.45	0.58
Total	KPR %	5.10	4.72	4.83	3.91	0.28	0.05	0.01	0.33
	SCD	4.88	4.71	5.21	4.99	0.16	0.05	0.21	0.88

The data are presented as the least square mean and standard error of the mean. [1]Methionine, choline, folate, vitamin B_{12}. [2]CON-OCM = Control (0.45kg/d) without one-carbon metabolite supplementation; CON+OCM = Control (0.45 kg/d) with one-carbon metabolite supplementation; RES-OCM = Restricted (-0.23 kg/d) without supplementation; RES+OCM = Restricted (-0.23 kg/d) with supplementation. [3]MFI = Main effect of feed intake levels; OCM = Main effect of one-carbon metabolite supplementation; Gain×SUP = Main effect of feed intake levels interaction with one-carbon metabolite supplementation. [4]SEM = Standard error of the mean. [5]Ki-67 positive ratio.[6]Spatial cell density×10000. Different lowercase letters indicate $p \leq 0.05$, and UPPERCASE letters indicate $0.05 < p \leq 0.10$.

Table S4. Pearson correlation coefficients for relationships between measured factors in fetal small intestine at 161 days of age.

		CND[2]	VPR	SVPR	KPR	SCD	SIW
**CON-OCM[1]**	CAD	-0.03	0.31*	0.38**	0.11	0.25	0.20
	CND		-0.00	-0.19	0.21	-0.19	0.00
	VPR			0.89**	-0.21	0.00	-0.00
	SVPR				-0.26†	0.10	-0.07
	KPR					-0.19	0.23
	SCD						-0.14
**CON+OCM**	CAD	-0.04	0.35**	0.43**	-0.07	0.40**	-0.27†
	CND		-0.19	-0.33*	-0.00	-0.48**	-0.12
	VPR			0.91**	-0.01	0.28*	0.27†
	SVPR				0.01	0.42**	0.20
	KPR					-0.19	0.05
	SCD						-0.17
**REC-OCM**	CAD	0.08	-0.07	0.04	0.41**	0.43**	0.16
	CND		-0.11	-0.14	0.30*	-0.30*	0.17
	VPR			0.96**	-0.12	0.05	-0.13
	SVPR				-0.11	0.13	-0.20
	KPR					-0.14	0.39**
	SCD						-0.21
**RES+OCM**	CAD	-0.12	0.30*	0.36**	-0.05	0.34*	-0.17
	CND		-0.23	-0.28†	-0.12	-0.39**	-0.14
	VPR			0.96**	0.14	0.45**	0.01
	SVPR				0.16	0.55**	0.13
	KPR					-0.1	0.39**
	SCD						0.00

[1]CON-OCM = Control (0.45kg/d) without one-carbon metabolite supplementation; CON+OCM = Control (0.45kg/d) with one-carbon metabolite supplementation; RES-OCM = Restricted (-0.23kg/d) without supplementation; RES+OCM = Restricted (-0.23kg/d) with supplementation. [2]CAD = Capillary area density.CND = Capillary number density×10000. VPR = VEGFR2 positivity ratio. SVPR = Spatial VEGFR2 positivity rate ×10000. KPR = Ki-67 positive ratio. SCD = Spatial cell density×10000. *$p \leqslant 0.05$. **$p \leqslant 0.01$. †$0.1 \geqslant p > 0.05$.
